# Two-Year Hypertension Incidence Risk Prediction in Populations in the Desert Regions of Northwest China: Prospective Cohort Study

**DOI:** 10.2196/68442

**Published:** 2025-03-12

**Authors:** Yinlin Cheng, Kuiying Gu, Weidong Ji, Zhensheng Hu, Yining Yang, Yi Zhou

**Affiliations:** 1 Zhongshan School of Medicine Sun Yat-sen University Guangzhou China; 2 School of Public Health Soochow University Suzhou China; 3 People's Hospital of Xinjiang Uygur Autonomous Region Urumqi China

**Keywords:** hypertension, desert, machine learning, deep learning, prevention, clinical applicability

## Abstract

**Background:**

Hypertension is a major global health issue and a significant modifiable risk factor for cardiovascular diseases, contributing to a substantial socioeconomic burden due to its high prevalence. In China, particularly among populations living near desert regions, hypertension is even more prevalent due to unique environmental and lifestyle conditions, exacerbating the disease burden in these areas, underscoring the urgent need for effective early detection and intervention strategies.

**Objective:**

This study aims to develop, calibrate, and prospectively validate a 2-year hypertension risk prediction model by using large-scale health examination data collected from populations residing in 4 regions surrounding the Taklamakan Desert of northwest China.

**Methods:**

We retrospectively analyzed the health examination data of 1,038,170 adults (2019-2021) and prospectively validated our findings in a separate cohort of 961,519 adults (2021-2023). Data included demographics, lifestyle factors, physical examinations, and laboratory measurements. Feature selection was performed using light gradient-boosting machine–based recursive feature elimination with cross-validation and Least Absolute Shrinkage and Selection Operator, yielding 24 key predictors. Multiple machine learning (logistic regression, random forest, extreme gradient boosting, light gradient-boosting machine) and deep learning (Feature Tokenizer + Transformer, SAINT) models were trained with Bayesian hyperparameter optimization.

**Results:**

Over a 2-year follow-up, 15.20% (157,766/1,038,170) of the participants in the retrospective cohort and 10.50% (101,077/961,519) in the prospective cohort developed hypertension. Among the models developed, the CatBoost model demonstrated the best performance, achieving area under the curve (AUC) values of 0.888 (95% CI 0.886-0.889) in the retrospective cohort and 0.803 (95% CI 0.801-0.804) in the prospective cohort. Calibration via isotonic regression improved the model’s probability estimates, with Brier scores of 0.090 (95% CI 0.089-0.091) and 0.102 (95% CI 0.101-0.103) in the internal validation and prospective cohorts, respectively. Participants were ranked by the positive predictive value calculated using the calibrated model and stratified into 4 risk categories (low, medium, high, and very high), with the very high group exhibiting a 41.08% (5741/13,975) hypertension incidence over 2 years. Age, BMI, and socioeconomic factors were identified as significant predictors of hypertension.

**Conclusions:**

Our machine learning model effectively predicted the 2-year risk of hypertension, making it particularly suitable for preventive health care management in high-risk populations residing in the desert regions of China. Our model exhibited excellent predictive performance and has potential for clinical application. A web-based application was developed based on our predictive model, which further enhanced the accessibility for clinical and public health use, aiding in reducing the burden of hypertension through timely prevention strategies.

## Introduction

Cardiovascular diseases are among the leading causes of chronic noncommunicable diseases worldwide and have become the primary cause of death globally [1]. Hypertension, a major preventable risk factor for cardiovascular disease, accounts for approximately 50% of all cardiovascular-related deaths globally [2]. In China, the prevalence of hypertension has been rising due to rapid urbanization, increasing affluence, and an aging population. Recent surveys estimate that approximately 244.5 million Chinese adults (23.2%) are affected by hypertension, with this number continuing to increase [3,4]. In northwestern China, populations residing in the Taklamakan Desert region face unique public health challenges, as harsh environmental conditions such as extreme temperature fluctuations, frequent sandstorms, particulate matter pollution, and limited greenspace are associated with an elevated prevalence of hypertension [5-7]. Given the increasing burden of hypertension, there is an urgent need for effective tools to identify high-risk individuals early and implement preventive measures.

Numerous hypertension risk prediction models have been developed using electronic health records based on a variety of methodological approaches, including traditional statistical techniques such as Cox regression and logistic regression, as well as machine learning and deep learning methods [8,9]. Although these models have demonstrated promising discriminatory power, several limitations persist. First, many studies have constructed models using small sample sizes or data from a single medical center, thereby limiting the external validity and generalizability of these models [10-14]. Second, a significant proportion of models lack validation on prospective datasets or independent external datasets, which impedes the comprehensive assessment of model robustness [12,15-18]. Additionally, some models incorporate an excessive number of variables or include variables that are not easily accessible in health examinations, thus hindering their practical application [19,20]. Finally, the majority of these models have been developed using data from European and American populations, thereby limiting their applicability to populations in other regions, including Asia.

Therefore, the objective of this study was to develop and prospectively validate a 2-year hypertension incidence risk prediction model by using health examination data collected from populations residing in 4 regions surrounding the Taklamakan Desert between 2019 and 2023. Our model aims to stratify individuals by their risk of developing hypertension, thus facilitating targeted prevention and early intervention strategies.

## Methods

### Data Source and Study Design

This retrospective cohort study utilized health examination data from adults collected between 2019 and 2021 at 1750 hospitals and community clinics across 4 regions surrounding the Taklamakan Desert of northwest China. Participants' health records were categorized into 3 components: personal and lifestyle information, standard physical examinations, and clinical measurements. Personal and lifestyle data included demographic details, personal and family medical histories, smoking and alcohol consumption habits, dietary patterns, and physical activity levels. Standard physical examinations recorded BMI, waist circumference, heart rate, and blood pressure, while clinical measurements assessed blood biochemical parameters.

**Figure 1:**
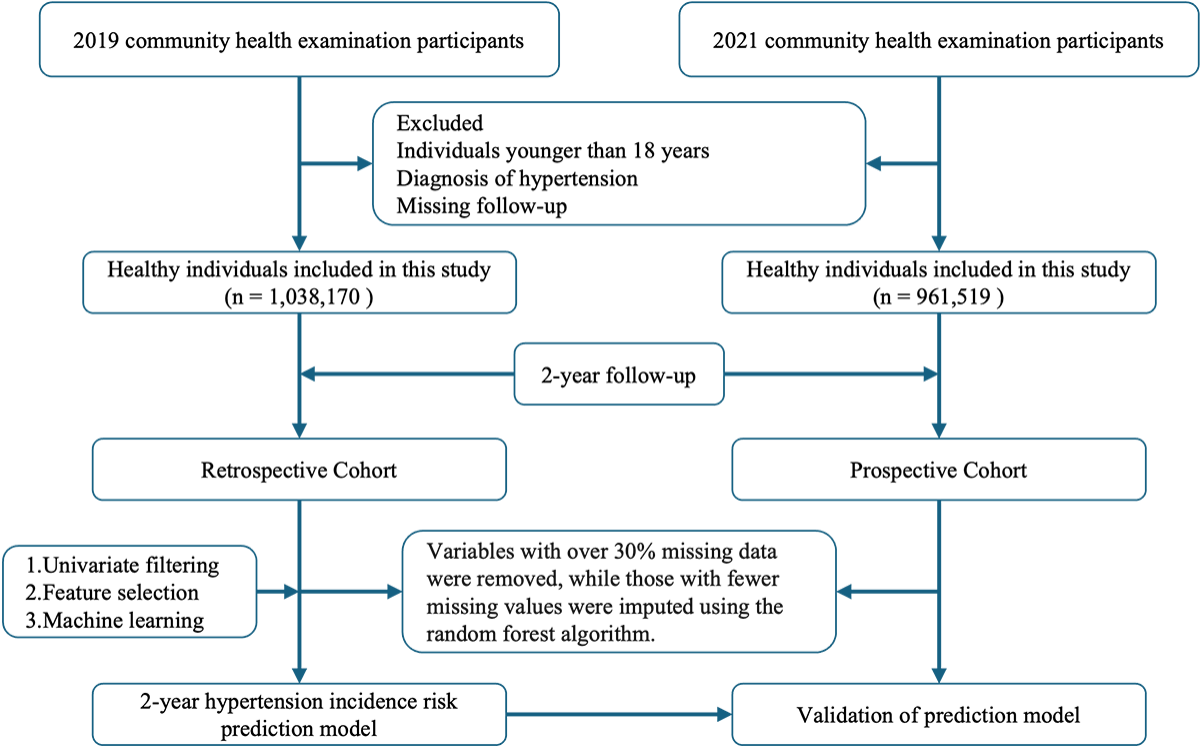
Flowchart illustrating the exclusion criteria, missing data handling, feature selection, model building (2019-2021), and validation (2021-2023) for the 2-year hypertension risk prediction study across 4 Taklamakan Desert–adjacent regions in northwest China.

A total of 2,872,837 participants were initially included. After applying the following exclusion criteria, that is, (1) individuals younger than 18 years, (2) individuals with a prior diagnosis of hypertension, and (3) participants with incomplete follow-up data, the final study cohort comprised 1,038,170 participants. For external validation, data from a prospective cohort (2021-2023) were employed, following the exclusion criteria outlined in Figure 1. Variables with more than 30% missing data were excluded from the analysis, following the methodology used in a previous study [21]. For variables with fewer missing values, the random forest algorithm was used for imputation. Multimedia Appendices 1 and 2 provide detailed information on the percentage of missing values for each variable in the retrospective and prospective cohorts prior to imputation.

### Ethics Approval

This study was approved by the Tsinghua University Science and Technology ethics committee (Medicine; project: 20240123). The data used in this research were collected and managed by the Xinjiang Uygur Autonomous Region Health Commission and were fully anonymized and deidentified before being accessed. According to ethical guidelines in China (Section 3.32), the secondary use of anonymized health data for research purposes is exempt from requiring additional informed consent from individual patients [22]. This exemption aligns with international research practices [23]. Researchers accessed the anonymized data through a designated, secure platform without internet connectivity, ensuring strict data privacy and security protocols. Neither the manuscript nor the supplementary materials contain identifiable information or images of participants, and no financial compensation was involved, as this study was based on secondary data analysis.

### Definition of Hypertension

Blood pressure was measured by trained health care professionals by using standardized protocols. The average of 3 consecutive right-arm measurements, taken 30 seconds apart, was used for analysis. Hypertension was defined by any of the following criteria: (1) self-reported diagnosis of hypertension, (2) current use of antihypertensive medication, or (3) an average systolic blood pressure ≥140 mm Hg, diastolic blood pressure ≥90 mm Hg, or both [24,25].

### Statistical Analysis

The Kolmogorov-Smirnov test was used to assess whether continuous variables followed a normal distribution. To compare baseline characteristics between participants with and without hypertension, we applied the chi-square test for categorical variables, while continuous variables were analyzed using either the independent-sample *t* test (2-sided) or the rank-sum test, depending on the distribution characteristics.

### Prediction Modeling and Evaluation

Participants from the retrospective cohort were randomly assigned to training and internal validation sets in a 7:3 ratio. Feature selection commenced with univariate logistic regression analyses to identify independent risk factors. Subsequently, both light gradient-boosting machine (LightGBM)–based recursive feature elimination with cross-validation (RFECV) [26,27] and Least Absolute Shrinkage and Selection Operator (LASSO) methods were applied to further refine the feature set. The final predictive features were determined by selecting the intersection of variables identified by both LightGBM-RFECV and LASSO.

The final model was built using CatBoost, a high-performance gradient boosting algorithm [28]. Additionally, several other models, including logistic regression, random forest, extreme gradient boosting [29], LightGBM, and 2 deep learning methods (Feature Tokenizer + Transformer [30] and SAINT [31]), were constructed to compare predictive performance for hypertension incidence. Bayesian optimization via *Hyperopt* [32] was employed to fine-tune the hyperparameters, maximizing the area under the receiver operating characteristic (AUROC) curve across 5-fold cross-validation. Each model underwent 1000 optimization trials. The detailed parameters for each model are provided in Multimedia Appendix 3.

The optimal model was evaluated using AUROC, average precision, accuracy, sensitivity, specificity, and confusion matrix in the internal validation set. Calibration of the selected model on the training set was performed using isotonic regression [33] to improve predictive accuracy and reliability. The calibrated model was subsequently transformed to both the internal validation set and the prospective cohort. Calibration curves, constructed with 25 evenly spaced bins, were then used to evaluate the model’s calibration and predictive performance across both validation sets.

To further refine risk stratification, we calculated the positive predictive values for individuals in the prospective cohort by using the calibrated model. Individuals were ranked by positive predictive values and categorized into 4 risk levels, ranging from low to very high risk. Univariate Cox regression was performed to validate the effectiveness of these risk categories.

### Model Interpretation

Shapley Additive Explanations (SHAP) [34] was employed to interpret the contribution of each feature to the model’s predictions. For highly weighted features such as age, gender, and BMI, we created subpopulations to analyze the distribution of risk categories within these groups. All analyses were conducted using Python software (version 3.8.0) with packages, including *CatBoost* (version 1.2.5), *Hyperopt* (version 0.2.7), and *SHAP* (version 0.44.1), as well as R software (version 4.0.2; R Foundation for Statistical Computing). A 2-tailed *P* value less than .05 was considered statistically significant.

## Results

### Clinical Baseline Characteristics

Table 1 outlines the baseline characteristics of the study cohorts. After applying the exclusion criteria, the retrospective cohort included 1,038,170 participants, aged 18-100 years, with baseline characteristics recorded between January and November 2019. The prospective cohort comprised 961,519 participants within the same age range, with baseline characteristics collected from January to November 2021. Over the 2-year follow-up period, the incidence of hypertension was 15.20% (157,766/1,038,170) in the retrospective cohort and 10.50% (101,077/961,519) in the prospective cohort. At baseline, compared to the nonhypertensive group, the hypertensive group had a higher proportion of males, resided in urban areas, and had lower education levels. The hypertensive group also had higher prevalence of hepatic steatosis and type 2 diabetes along with elevated waist circumference, BMI, systolic blood pressure, and diastolic blood pressure. Multimedia Appendices 4 and 5 provide a detailed comparison of the characteristics between individuals with and without incident hypertension in both cohorts.

**Table 1:** Clinical baseline information of the retrospective and prospective cohorts^a^.

Characteristics			Retrospective cohort (n=1,038,170)		Prospective cohort (n=961,519)
Age at baseline (years), mean (SD)			40.11 (14.73)		42.77 (14.45)
**Sex, n (%)**					
	Male	458,964 (44.21)		431,824 (44.91)	
	Female	579,206 (55.79)		529,695 (55.09)	
**Residence, n (%)**					
	Rural	915,254 (88.16)		865,464 (90.01)	
	Urban	122,916 (11.84)		96,055 (9.99)	
Waist circumference (cm), mean (SD)			83.37 (10.65)		85.00 (11.21)
BMI (kg/m^2^), mean (SD)			23.80 (3.67)		24.64 (3.82)
**Educational level, n** **(%)**					
	Illiterate or semiliterate	42,801 (4.12)		40,876 (4.25)	
	Primary school	381,811 (36.78)		376,961 (39.2)	
	Junior middle school	480,768 (46.31)		410,493 (42.69)	
	Senior middle school	87,775 (8.45)		85,923 (8.94)	
	College degree and above	45,015 (4.34)		47,266 (4.92)	
**Exercise frequency, n** **(%)**					
	Never	1,000,327 (96.35)		922,358 (95.93)	
	Occasionally	7592 (0.73)		16,839 (1.75)	
	Often	30,251 (2.92)		22,322 (2.32)	
**Dietary patterns, n (%)**					
	Meat and vegetable balanced	939,046 (90.45)		936,106 (97.36)	
	Meat-based	52,539 (5.06)		13,578 (1.41)	
	Vegetarian-based	46,585 (4.49)		11,835 (1.23)	
**Smoking status, n** **(%)**					
	Never	889,175 (85.65)		861,763 (89.63)	
	Smoking	137,212 (13.22)		96,374 (10.02)	
	Quit smoking	11,783 (1.13)		3382 (0.35)	
**Alcohol intake, n** **(%)**					
	Never	923,357 (88.94)		894,674 (93.05)	
	Occasionally	102,187 (9.84)		60,485 (6.29)	
	Often	12,626 (1.22)		6360 (0.66)	
Heart rate (bpm), mean (SD)			73.59 (10.13)		74.30 (9.41)
Systolic blood pressure (mm Hg), mean (SD)			109.03 (12.25)		111.55 (11.66)
Diastolic blood pressure (mm Hg), mean (SD)			66.41 (8.45)		67.81 (8.01)
Hemoglobin (g/L), mean (SD)			139.76 (17.51)		141.38 (17.47)
White blood cell count (10^9^/L), mean (SD)			6.40 (1.44)		6.44 (1.49)
Alanine aminotransferase (U/L), mean (SD)			21.07 (9.02)		20.94 (9.20)
Aspartate transaminase (U/L), mean (SD)			22.27 (6.51)		21.90 (6.81)
Serum creatinine (μmol/L), mean (SD)			66.08 (17.64)		66.59 (17.78)
Total cholesterol (mmol/L), mean (SD)			4.07 (0.87)		4.14 (0.90)
HDL-C^a^ (mmol/L), mean (SD)			1.31 (0.33)		1.28 (0.34)
LDL-C^b^ (mmol/L), mean (SD)			2.32 (0.75)		2.25 (0.84)
**Hepatic steatosis, n (%)**					
	No	999,729 (96.3)		932,220 (96.95)	
	Yes	38,441 (3.7)		29,299 (3.05)	
**Type 2 diabetes, n (%)**					
	No	1,002,725 (96.59)		921,947 (95.88)	
	Yes	35,445 (3.41)		39,572 (4.12)	
**Family history of hypertension, n** **(%)**					
	No	952,605 (91.76)		883,277 (91.86)	
	Yes	85,565 (8.24)		78,242 (8.14)	

^a^HDL-C: high-density lipoprotein cholesterol.

^b^LDL-C: low-density lipoprotein cholesterol.

### Development and Validation of Predictive Models

**Figure 2:**
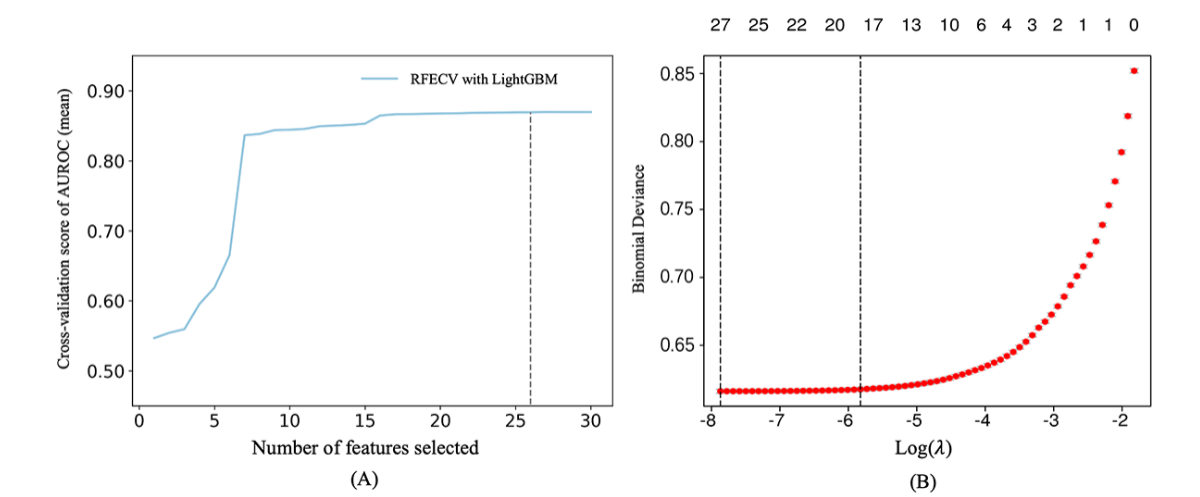
Feature selection of the model. (A) Feature selection results based on light gradient-boosting machine–based recursive feature elimination with cross-validation. (B) Feature selection results based on Least Absolute Shrinkage and Selection Operator. AUROC: area under the receiver operating characteristic; LightGBM: light gradient-boosting machine; RFECV: recursive feature elimination with cross-validation.

Recursive feature elimination is a widely used method for feature selection. It iteratively trains a model, assesses the importance of each feature, and removes the least important ones until the optimal number of features is identified. In this study, we used an enhanced version of recursive feature elimination, RFECV, which incorporates cross-validation to evaluate model performance at each iteration. Additionally, LASSO regression was employed, using L1 regularization to shrink the coefficients of less important features to zero, thereby further refining the feature set (Figure 2). Through the application of both LightGBM-RFECV and LASSO, we identified 26 and 27 potential risk predictors for hypertension, respectively. By intersecting the results of these 2 methods, we selected 24 key features for the final construction of the 2-year hypertension incidence risk prediction model.

### Model Performance

**Figure 3:**
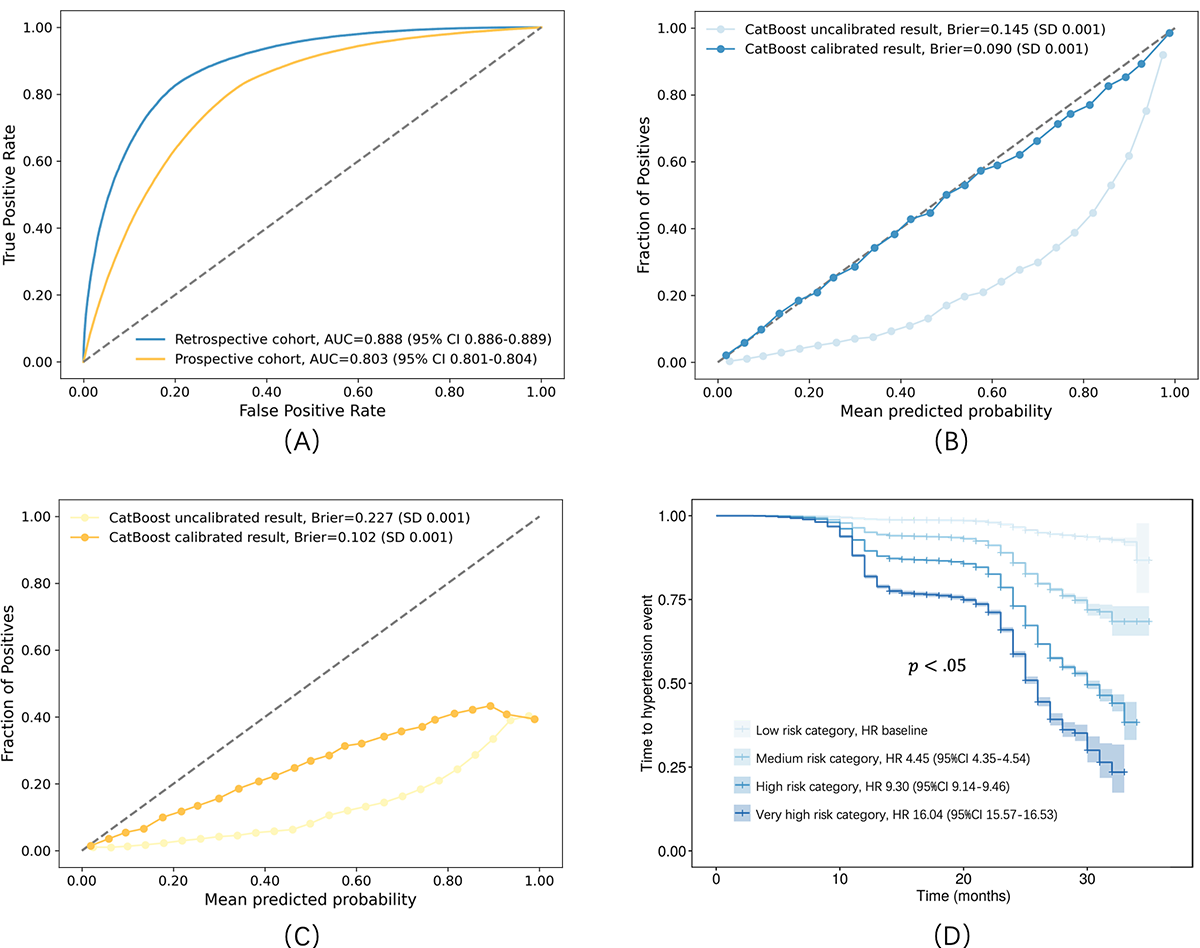
(A) Receiver operating characteristic curve of the CatBoost model in the retrospective and prospective cohorts. (B) Noncalibrated and calibrated curves of the CatBoost model in the retrospective cohort. (C) Noncalibrated and calibrated curves of the CatBoost model in the prospective cohort. (D) Survival curves for time-to-hypertension across 4 risk categories. AUC: area under the curve; HR: hazard ratio.

We evaluated the performance of several machine learning and deep learning models. Among the models, CatBoost demonstrated the highest predictive accuracy, with an AUROC curve of 0.888 (95% CI 0.886-0.889). Extreme gradient boosting followed with an AUROC of 0.868 (95% CI 0.866-0.870). Detailed AUROC comparisons for each model are available in Multimedia Appendix 6, while the confusion matrix results are provided in Multimedia Appendix 7. A Mann-Whitney *U* test confirmed that CatBoost significantly outperformed the other algorithms in terms of predictive accuracy (*P*<.001) (Multimedia Appendix 8) [35]. Further validation of the CatBoost model was performed using the prospective cohort. Compared with the results from the internal validation set, the AUROC for the prospective cohort decreased to 0.803 (95% CI 0.801-0.804) (Figure 3). Isotonic regression was applied to calibrate the output probabilities, improving the accuracy of predicted probabilities in reflecting actual risks. After calibration, the model’s performance metrics improved, with a Brier score of 0.090 (95% CI 0.089-0.091) on the internal validation set and 0.102 (95% CI 0.101-0.103) on the prospective cohort.

Following calibration, we calculated the positive predictive values for each individual and categorized participants into 4 risk levels: low, medium, high, and very high. In the low-risk group (risk score 0-0.15, n=570,742), only 2.87% (16,392/570,742) developed hypertension over the 2-year period. In contrast, 41.08% (5741/13,975) of the individuals in the very high-risk group (risk score 0.8-1, n=13,975) were diagnosed with hypertension within the same period (Multimedia Appendix 9). To further assess the differences in hypertension onset across the 4 risk categories, we performed univariate Cox regression to estimate time-to-hypertension for each group. The results showed significant differences between the risk categories (*P*<.05), with the hazard ratio for the very high-risk group being 16.4 (95% CI 15.57-16.53) compared with the low-risk group.

### Feature Importance and SHAP Analysis

**Figure 4:**
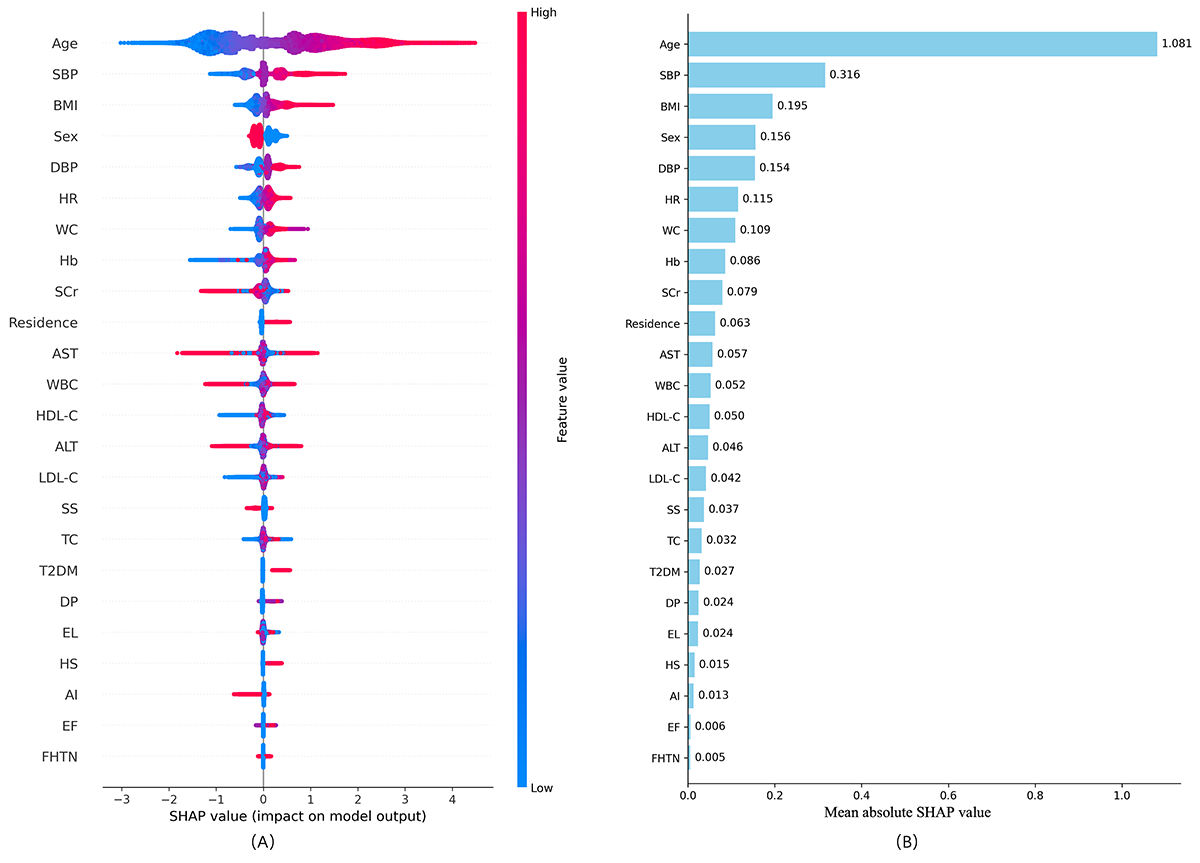
(A) Complete distribution of Shapley Additive Explanations values for all features in the prospective dataset. (B) Absolute contribution of all features based on Shapley Additive Explanations values for all features in the prospective dataset. AI: alcohol intake; ALT: alanine aminotransferase; AST: aspartate transaminase; DBP: diastolic blood pressure; DP: dietary pattern; EF: exercise frequency; EL: educational level; FHTN: family history of hypertension; Hb: hemoglobin; HDL-C: high-density lipoprotein cholesterol; HR: heart rate; HS: hepatic steatosis; LDL-C: low-density lipoprotein cholesterol; SBP: systolic blood pressure; SCr: serum creatinine; SHAP: Shapley Additive Explanations; SS: smoking status; T2DM: type 2 diabetes; TC: total cholesterol; WBC: white blood cell; WC: waist circumference.

We used the distribution of SHAP values and the corresponding mean absolute SHAP values within the prospective cohort to interpret the contribution of each feature in the risk prediction model. The analysis revealed that the model’s core predictive power derived primarily from basic biometrics (age, BMI, gender, and waist circumference), blood pressure indicators (systolic and diastolic blood pressure), and heart rate (Figure 4).

**Figure 5:**
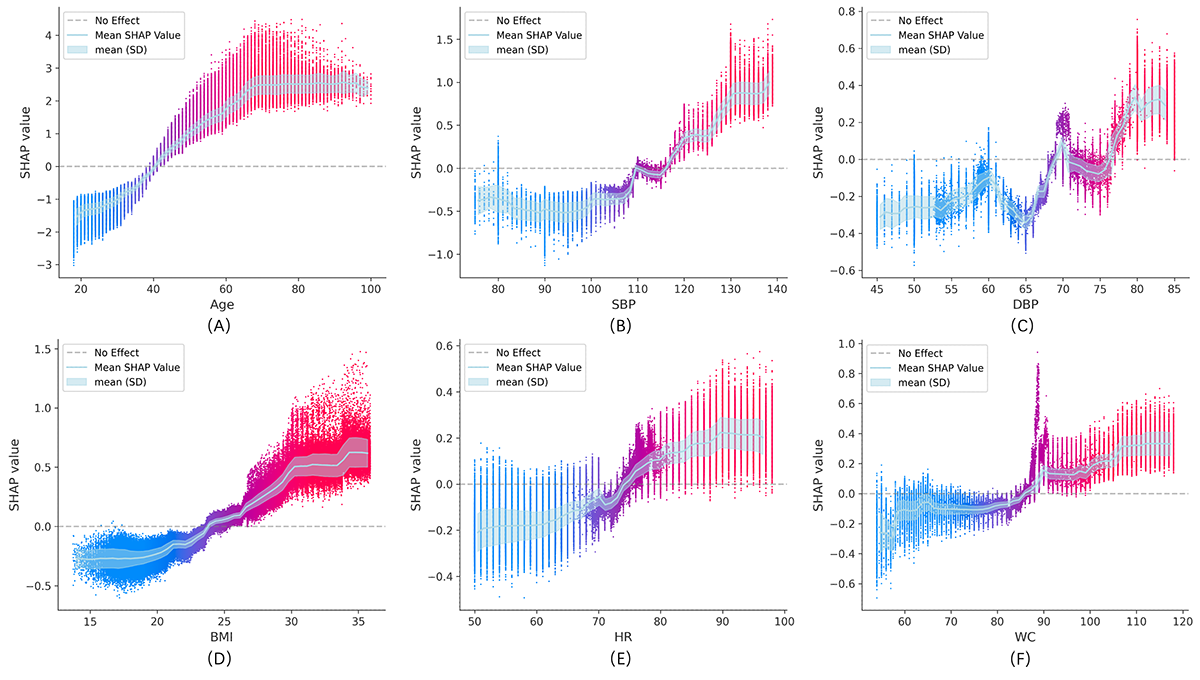
(A)-(F) Shapley Additive Explanations value dependence plots for 6 example features in the prospective dataset. DBP: diastolic blood pressure; HR: heart rate; SBP: systolic blood pressure; SHAP: Shapley Additive Explanations; WC: waist circumference.

To better understand the impact of individual variables, we analyzed the SHAP values for the top 6 ranked continuous variables (Figure 5). We observed that as these feature values increased, their SHAP values transitioned from negative to positive, indicating that higher values significantly increased the likelihood of being classified as high-risk for hypertension.

### Significant Features

In addition to analyzing SHAP values for individual features, we conducted a grouped analysis of the most influential basic biometrics (age, sex, and BMI) and socioeconomic factors (residence and education level) within the prospective cohort to assess the distribution of the samples across different risk levels and the model’s predictive performance within these feature groups.

**Figure 6:**
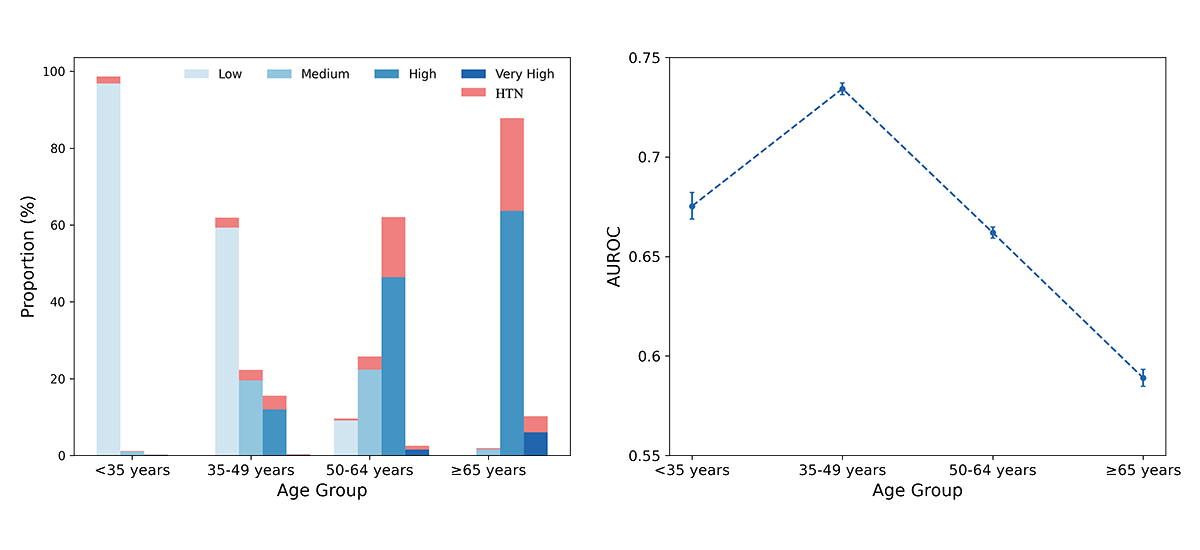
(A) Proportion of 4 risk categories across different age group subsets. (B) Area under the receiver operating characteristic curve values evaluated for different age group subsets. AUROC: area under the receiver operating characteristic curve; HTN: hypertension.

Based on prior research [19], we divided the population into 4 age categories. The analysis showed that the young population was predominantly in the low-risk category (343,394/347,996, 98.68%), while the older adult population was primarily composed of high-risk (68,238/77,664, 87.86%) and very high-risk (7939/77,664, 10.22%) individuals (Figure 6). Hypertension incidence within 2 years in the older adults (22,277/77,664, 28.68%) was significantly higher than that in the younger population (7154/347,996, 2.05%), confirming age as a key predictor of hypertension onset. Further analysis (Figure 6B) demonstrated that the model performed best in the 35-49 years age group, with an AUROC of 0.734 (95% CI 0.732-0.737), whereas performance was relatively weaker in the older adult population, with an AUROC of 0.589 (95% CI 0.585-0.593). This performance disparity may be attributed to the model’s tendency to assign higher risk to older individuals, reducing its discriminatory power in this group.

**Figure 7:**
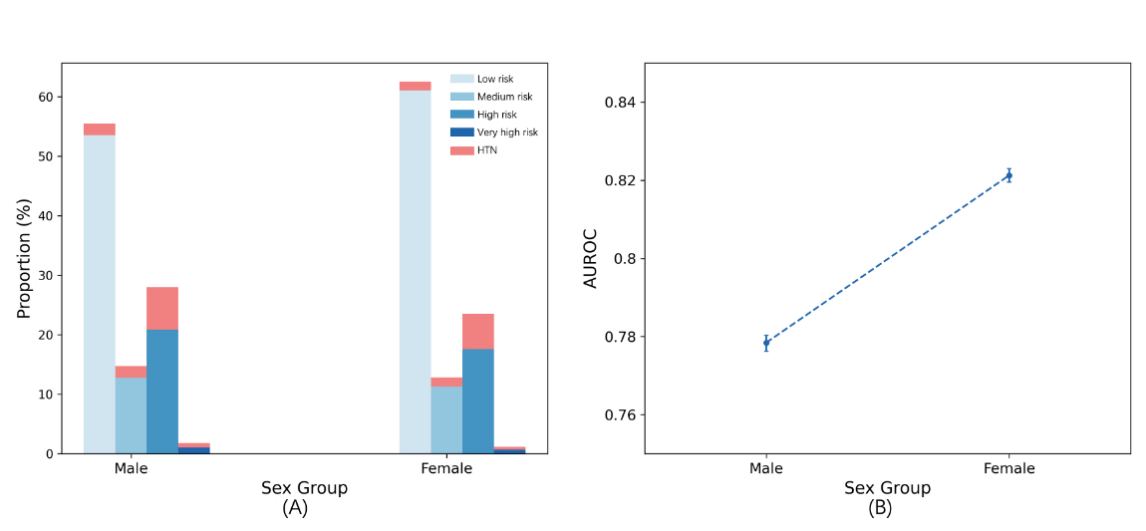
(A) Proportion of 4 risk categories across different sex group subsets. (B) Area under the receiver operating characteristic curve values evaluated for male and female subgroups. AUROC: area under the receiver operating characteristic curve; HTN: hypertension.

Regarding gender, hypertension incidence within 2 years in women (49,855/529,695, 9.41%) was lower than that in men (51,222/431,824, 11.86%). A higher proportion of women were in the low-risk category, that is, 62.51% (331,122/529,695) of the women were in the low-risk category, while only 55.49% (239,620/431,824) of the men were in the low-risk category (Figure 7). Conversely, men were more likely to be in the high-risk (120,676/431,824, 27.95%) and very high-risk (7821/431,824, 1.81%) categories. Additionally, the model performed better in women, achieving an AUROC of 0.821 (95% CI 0.820-0.823) compared to men with an AUROC of 0.785 (95% CI 0.777-0.781).

**Figure 8:**
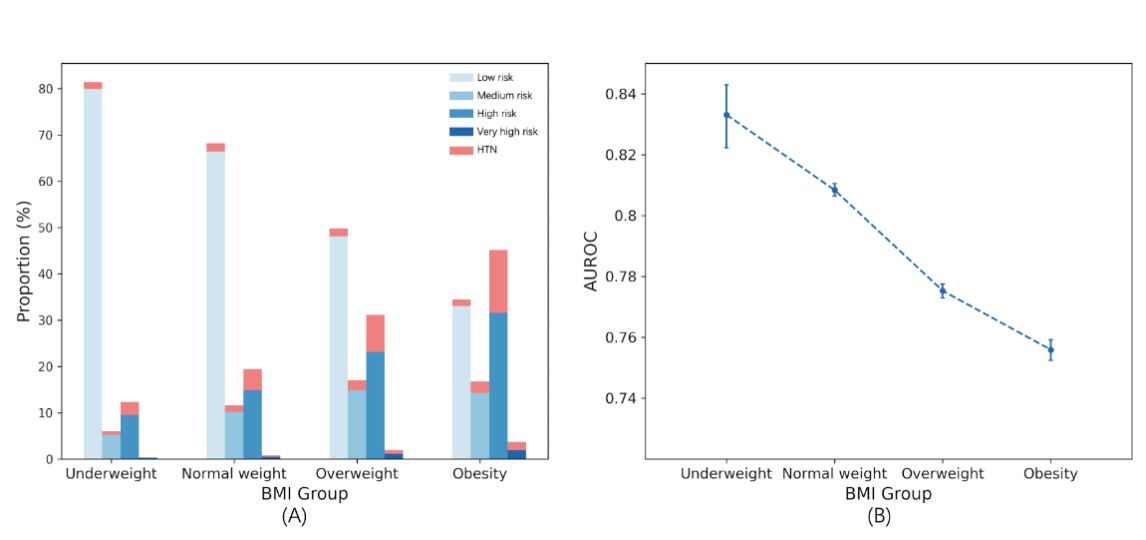
(A) Proportion of 4 risk categories across different BMI group subsets. (B) Area under the receiver operating characteristic curve; values evaluated for different BMI group subsets. AUROC: area under the receiver operating characteristic curve; HTN: hypertension.

BMI was categorized according to the World Health Organization classification: underweight (BMI<18.5), normal weight (18.5≤BMI<25), overweight (25≤BMI<30), and obesity (BMI≥30). As BMI increased, the proportion of individuals in the high-risk and very high-risk categories also increased along with a corresponding increase in hypertension incidence. This indicates a strong association between obesity and hypertension risk (Figure 8).

**Figure 9:**
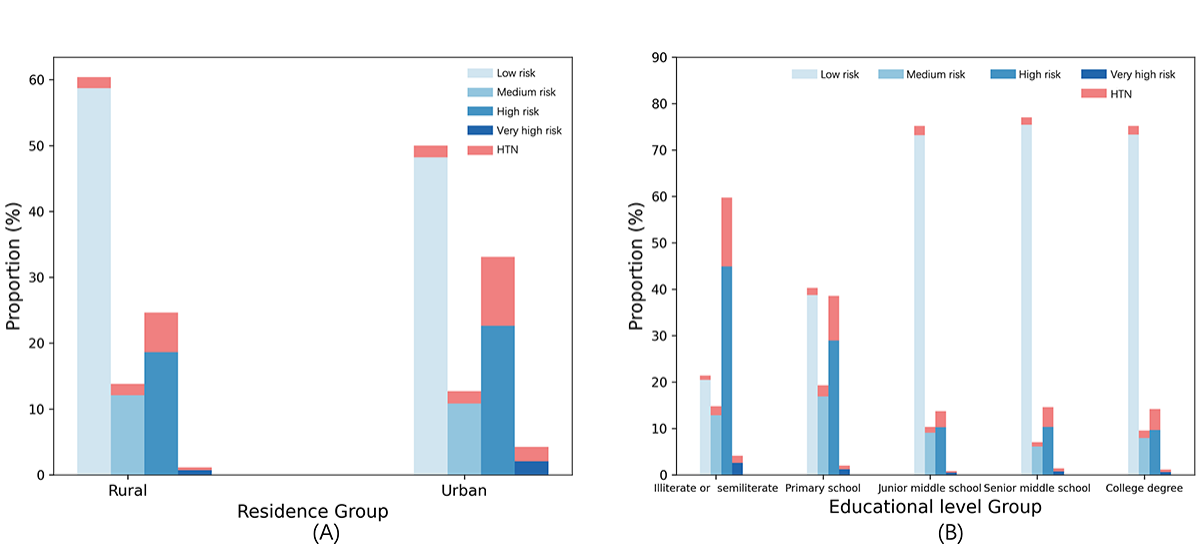
(A) Proportion of 4 risk categories across different residence group subsets. (B) Proportion of 4 risk categories across different educational level group subsets. HTN: hypertension.

We also analyzed the impact of socioeconomic factors on hypertension risk (Figure 9). Among urban residents, the proportion of individuals classified as high risk (31,764/96,055, 33.07%) and very high-risk (4084/96,055, 4.25%) was greater than that among rural residents, where the high-risk group accounted for 24.66% (213,441/865,464) and the very high-risk group for 1.14% (9891/865,464). Hypertension incidence among the urban population (15,664/96,055, 16.31%) was also higher than that among rural residents (85,414/865,464, 9.87%). Similarly, individuals with lower levels of education (illiterate or semiliterate, primary school) had higher proportions in the high-risk (169,594/417,837, 40.59%) and very high-risk (9104/417,837, 2.18%) categories, with correspondingly higher incidence rates (61,741/417,837, 14.77%). These findings suggest that socioeconomic factors such as living environment and education level are closely linked to hypertension risk.

Finally, we developed a web-based application based on our predictive model (see main interface in Multimedia Appendix 10) [36], designed for integration into routine clinical workflows. During health examinations, clinicians can input patient data into the application, which then instantly generates individualized hypertension risk assessments. Each patient is assigned to 1 of the 4 actionable risk levels (low, medium, high, very high), aiding in targeted clinical decision-making. The application also employs interpretable SHAP value visualizations, allowing clinicians to understand the key contributors to each patient’s risk profile. The application is publicly accessible to encourage external validation by health care providers [36], and we have also released the corresponding open-source code (Multimedia Appendix 11) [37].

## Discussion

### Principal Findings

This study shows a 2-year hypertension risk prediction model using a retrospective cohort of over 1 million participants from populations residing in the desert regions of China, with both internal and external validation. Using feature selection techniques, we identified 24 significant variables. The CatBoost model demonstrated superior predictive performance, achieving an area under the curve (AUC) of 0.888 in the retrospective cohort and 0.803 in the prospective cohort, outperforming other machine learning and deep learning models. During the 2-year follow-up in the prospective cohort, the model effectively stratified individuals into 4 distinct risk categories, revealing significant differences in the hazard ratios for hypertension incidence among these groups. Analysis of the model’s significant features indicated that the risk of hypertension is strongly influenced by basic biometrics (age, sex, and BMI) and socioeconomic factors (residence and education level). Furthermore, a web-based application was developed and made open-source, providing clinicians with a practical and accessible tool to assess hypertension risk and guide early intervention strategies.

According to the World Health Organization’s report on global hypertension prevalence, approximately 1.1 billion adults aged 30-79 years have hypertension, with two-thirds residing in low- and middle-income countries [25]. China, as the largest middle-income country, has an estimated hypertension prevalence of 27.5% [38]. In this study, we observed an incidence rate of 56.30 per 1000 person-years, which is higher than the national average of 48.60 per 1000 person-years reported between 2011 and 2015 [39]. This discrepancy may be attributed to the geographic location of our cohort, composed of residents from 4 regions near the Taklamakan Desert in northwest China. Environmental factors, lifestyle choices, and dietary patterns specific to this area likely contributed to the higher incidence, underscoring a more severe public health challenge in these regions and highlighting the need for targeted prevention strategies.

CatBoost was chosen as the hypertension risk prediction model due to its superior performance compared to other gradient-boosting decision tree models, such as LightGBM and extreme gradient boosting. CatBoost’s key advantage lies in its ability to efficiently handle high-dimensional categorical data, thanks to its unique target encoding and ordered boosting algorithms. These methods minimize data leakage and prevent the use of future information in current predictions, enhancing model generalizability and reducing the need for extensive preprocessing. Although recent advancements in deep learning for tabular data prediction have shown promise, the 2 deep learning models (Feature Tokenizer + Transformer and SAINT) evaluated in this study underperformed slightly compared to CatBoost. Feature Tokenizer + Transformer leverages feature tokenization, transforming features into tokens for the Transformer architecture, while SAINT introduces intersample attention mechanisms to capture relationships between samples. Despite their slightly lower performance in this dataset, these models demonstrated comparable results to other gradient-boosting decision tree models, indicating that deep learning approaches have strong potential for future application in tabular data prediction.

When contrasted with previous studies [10-16], this study represents an advancement in both sample size and model performance. Our dataset included data from 1750 hospitals and community clinics, encompassing a total of 2 million individuals, ensuring the reliability and robustness of the results. The CatBoost model achieved an AUC of 0.888 in the retrospective cohort and 0.803 in the prospective cohort. By comparison, López-Martínez et al [16] utilized the 2007-2016 National Health and Nutrition Examination Survey dataset to develop 2 predictive models: a logistic regression model reported in 2018, which achieved an internal validation AUC of 0.73 [15] and an artificial neural network model developed in 2020, which improved the AUC to 0.77.

Consistent with prior studies, our model identified age, gender, and BMI as the major predictors of hypertension [3,40,41]. We found that as age and BMI increased, the proportion of individuals in the high-risk category rose significantly, further emphasizing their importance in hypertension risk prediction. In terms of socioeconomic factors, our study showed that individuals residing in urban areas and those with lower education levels had higher incidences of hypertension over 2 years. This may be due to urban populations engaging in consuming diets high in salt and fat while lacking overall nutritional quality, less physical activity, and being exposed to more severe environmental pollution [42-44], while individuals with lower education levels may have reduced access to health care and limited health literacy, both of which increase their vulnerability to hypertension [45].

The clinical implications of this study are significant. To our knowledge, this is one of the first large-scale, prospective cohort studies focused on hypertension risk prediction in populations residing in regions surrounding a desert. The model relies on 24 basic clinical and biochemical features that are routinely collected during health checkups, making it practical for use in primary health care settings. Given the limited health care resources in desert regions, the model can assist health care providers in identifying high-risk individuals for hypertension up to 2 years in advance. This allows for optimized medical decision-making and resource allocation, promoting earlier intervention and potentially reducing the overall disease burden.

Furthermore, the development of a web-based application extends the model’s utility, allowing for broader clinical implementation. This user-friendly tool provides an intuitive way for clinicians to assess hypertension risk predictions and the specific contributions of individual risk factors. Especially in resource-constrained regions, this model offers an opportunity for precision prevention and intervention, potentially reducing long-term health care costs and improving patient outcomes. These contributions collectively aim to address previous methodological and contextual limitations, expand the body of knowledge, and provide potential solutions to pressing public health challenges.

### Limitations

This study has several important limitations that may affect the interpretation and generalizability of the findings. First, some of the health examination data contained missing values. Although we addressed this issue by using a random forest imputation method based on the training set, this approach may introduce bias, particularly if the missing data are not missing completely at random. Imputation can improve data completeness, but it may still impact the accuracy of the model’s predictions and its external validity. Second, the range of variables included in this study was limited. Important factors such as psychological stress, genetic predisposition, and environmental influences were not incorporated into the model. These factors have been shown to contribute significantly to hypertension risk, and their omission may limit the comprehensiveness of the model. In addition, the model was developed using data from specific geographic region populations surrounding desert areas in northwest China. Although this provides valuable insights for these communities, the model’s applicability to other populations, particularly those in different geographic and socioeconomic settings, may be limited. Lastly, certain lifestyle factors such as physical activity and diet were based on self-reported data, which are subject to recall bias and may not accurately reflect actual behaviors.

### Conclusions

In conclusion, we developed and validated a 2-year hypertension incidence risk prediction model by using data from nearly 2 million individuals residing in regions surrounding the Taklamakan Desert. The model demonstrated strong predictive performance, with high accuracy in both retrospective and prospective cohorts. By stratifying individuals into distinct risk categories, the model identified significant variations in hypertension incidence rates between these groups, underscoring its potential as a valuable tool for risk stratification in clinical practice. To enhance clinical applicability, we further developed a web-based hypertension prediction application, facilitating early screening and intervention for high-risk populations. This study not only holds significant clinical value but also provides an efficient public health tool for regions with limited health care resources, supporting early prevention and precision management of hypertension.

## Appendix

Multimedia Appendix 1 Distribution of missing values across variables in the retrospective cohort.

Multimedia Appendix 2 Distribution of missing values across variables in the prospective cohort.

Multimedia Appendix 3 Optimal hyperparameter for each model.

Multimedia Appendix 4 Baseline characteristics of the study population in the retrospective cohort.

Multimedia Appendix 5 Baseline characteristics of the study population in the prospective cohort.

Multimedia Appendix 6 Evaluation of multiple machine learning models to predict the 2-year hypertension incidence risk.

Multimedia Appendix 7 Confusion matrices of multiple machine learning models for predicting the 2-year hypertension incidence risk.

Multimedia Appendix 8 Statistical significance analysis of area under the receiver operating characteristic curve comparisons between CatBoost and other models.

Multimedia Appendix 9 Classification of individuals into 4 risk levels based on the positive predictive values in the prospective dataset.

Multimedia Appendix 10 Main interface of the web-based application for 2-year hypertension incidence risk prediction.

Multimedia Appendix 11 Hypertension risk prediction web application.

## Abbreviations

AUC: area under the curve
AUROC: area under the receiver operating characteristic
LASSO: Least Absolute Shrinkage and Selection Operator
LightGBM: light gradient-boosting machine
RFECV: recursive feature elimination with cross-validation
SHAP: Shapley Additive Explanations

## Data Availability

The datasets generated during and analyzed during this study are available from the corresponding author on reasonable request.
