# Supplementary material for: Two-Year Hypertension Incidence Risk Prediction in Populations in the Desert Regions of Northwest China: Prospective Cohort Study
Source: J Med Internet Res. 2025 Mar 12;27:e68442. doi: 10.2196/68442 (PMC11947627; doi:10.2196/68442)
Supplement: Multimedia Appendix 1 [file jmir_v27i1e68442_app1.pdf]

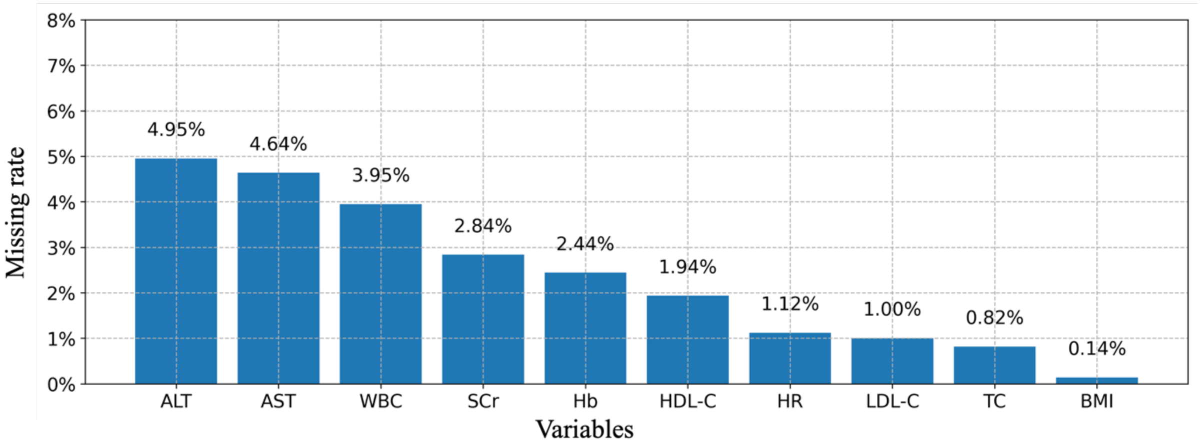

Multimedia Appendix 1. Distribution of missing values across variables in the retrospective cohort. Abbreviations: ALT, alanine aminotransferase; AST, aspartate transaminase; WBC, white blood cell; SCr, serum creatinine; Hb, hemoglobin; HDL-C, high-density lipoprotein cholesterol; HR, heart rate; LDL-C, low-density lipoprotein cholesterol; TC, total cholesterol; BMI, body mass index.
