# Supplementary material for: Two-Year Hypertension Incidence Risk Prediction in Populations in the Desert Regions of Northwest China: Prospective Cohort Study
Source: J Med Internet Res. 2025 Mar 12;27:e68442. doi: 10.2196/68442 (PMC11947627; doi:10.2196/68442)
Supplement: Multimedia Appendix 2 [file jmir_v27i1e68442_app2.pdf]

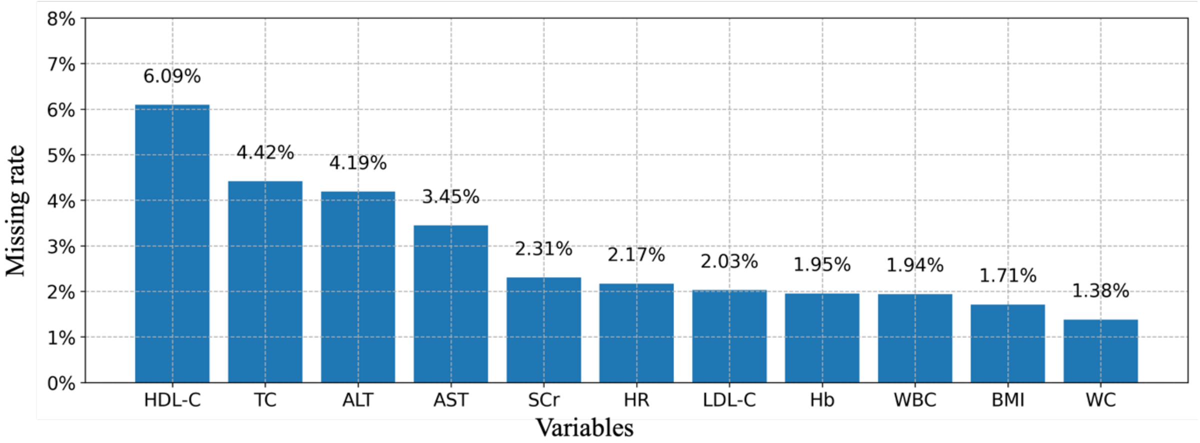

Multimedia Appendix 2. Distribution of missing values across variables in the prospective cohort. Abbreviations: HDL-C, high-density lipoprotein cholesterol; TC, total cholesterol; ALT, alanine aminotransferase; AST, aspartate transaminase; SCr, serum creatinine; HR, heart rate; LDL-C, low-density lipoprotein cholesterol; Hb, hemoglobin; WBC, white blood cell; BMI, body mass index, WC, waist circumference.
