# Supplementary material for: Two-Year Hypertension Incidence Risk Prediction in Populations in the Desert Regions of Northwest China: Prospective Cohort Study
Source: J Med Internet Res. 2025 Mar 12;27:e68442. doi: 10.2196/68442 (PMC11947627; doi:10.2196/68442)
Supplement: Multimedia Appendix 3 [file jmir_v27i1e68442_app3.pdf]

**Multimedia Appendix 3.** Optimal hyperparameter for each model

| Model | Hyperparameter | Value |
|---|---|---|
| Logistic Regression | solver | sag |
| | max_iter | 300 |
| | C | 0.8 |
| Random Forest | max_depth | 70 |
| | max_features | log2 |
| | bootstrap | True |
| | criterion | entropy |
| LightGBM | learning_rate | 0.060000000000000005 |
| | min_child_weight | 7 |
| | max_depth | 5 |
| | colsample_bytree | 0.4 |
| | subsample | 0.9742705680298589 |
| | n_estimators | 500 |
| | num_leaves | 70 |
| XGBoost | learning_rate | 0.01 |
| | gamma | 0.02 |
| | min_child_weight | 7 |
| | max_depth | 8 |
| | colsample_bytree | 0.7000000000000002 |
| | subsample | 0.8193311950361888 |
| | n_estimators | 1000 |
| CatBoost | learning_rate | 0.042433385590018684 |
| | depth | 7 |
| | iterations | 1400 |
| | rsm | 0.5997087570432558 |
| | l2_leaf_reg | 25.57667048362044 |
| | bootstrap_type | MVS |
| | random_strength | 0.3114475735294593 |
| | subsample | 0.8735275260613191 |
| FT-Transformer | n_blocks | 6 |
| | d_block | 384 |
| | attention_n_heads | 8 |
| | n_epochs | 200 |
| | batch_size | 5120 |
| | lr | 0.001 |
| SAINT | cont_embeddings | MLP |
| | embedding_size | 32 |
| | attentiontype | colrow |
| | lr | 0.001 |
| | epochs | 100 |
| | batchsize | 512 |
