# Supplementary material for: Two-Year Hypertension Incidence Risk Prediction in Populations in the Desert Regions of Northwest China: Prospective Cohort Study
Source: J Med Internet Res. 2025 Mar 12;27:e68442. doi: 10.2196/68442 (PMC11947627; doi:10.2196/68442)
Supplement: Multimedia Appendix 4 [file jmir_v27i1e68442_app4.pdf]

Multimedia Appendix 4. Baseline characteristics of the study population in retrospective cohort

Characteristics	Overall	Non-HTN	HTN	p-value
	1,038,170	880,404	157,766	
Age at baseline, years	40.11 (14.73)	37.30 (12.89)	55.78 (14.53)	<0.001
Sex (%)				<0.001
male	458,964 (44.21)	379,256 (43.08)	79,708 (50.52)	
female	579,206 (55.79)	501,148 (56.92)	78,058 (49.48)	
Residence (%)				<0.001
rural	915,254 (88.16)	782,231 (88.85)	133,023 (84.32)	
urban	122,916 (11.84)	98,173 (11.15)	24,743 (15.68)	
WC, cm	83.37 (10.65)	82.57 (10.37)	87.80 (11.08)	<0.001
BMI, kg/m^2	23.80 (3.67)	23.57 (3.59)	25.07 (3.87)	<0.001
Educational level (%)				<0.001
illiterate or semi-literate	42,801 (4.12)	26,833 (3.05)	15,968 (10.12)	
primary school	381,811 (36.78)	294,910 (33.5)	86,901 (55.08)	
junior middle school	480,768 (46.31)	439,261 (49.89)	41,507 (26.31)	
senior middle school	87,775 (8.45)	79,089 (8.98)	8,686 (5.51)	
college degree and above	45,015 (4.34)	40,311 (4.58)	4,704 (2.98)	
Exercise frequency (%)				<0.001
never	1,000,327(96.35)	849,766 (96.52)	150,561 (95.43)	
occasionally	7,592 (0.73)	5,935 (0.68)	1,657 (1.05)	
often	30,251 (2.92)	24,703 (2.8)	5,548 (3.52)	
Dietary patterns (%)				<0.001
meat and vegetable balance	939,046 (90.45)	800,001 (90.87)	139,045 (88.13)	
meat based	52,539 (5.06)	42,931 (4.88)	9,608 (6.09)	
vegetarian based	46,585 (4.49)	37,472 (4.25)	9,113 (5.78)	
Smoking status (%)				<0.001
never	889,175 (85.65)	749,740 (85.16)	139,435 (88.38)	
smoking	137,212 (13.22)	121,328 (13.78)	15,884 (10.07)	
quit smoking	11,783 (1.13)	9,336 (1.06)	2,447 (1.55)	
Alcohol intake (%)				<0.001
never	923,357 (88.94)	778,022 (88.37)	145,335 (92.12)	
occasionally	102,187 (9.84)	90,904 (10.33)	11,283 (7.15)	
often	12,626 (1.22)	11,478 (1.3)	1,148 (0.73)	
Heart rate, bpm	73.59 (10.13)	73.39 (10.08)	74.73 (10.32)	<0.001
SBP, mmHg	109.03 (12.25)	107.71 (11.88)	116.42 (11.62)	<0.001
DBP, mmHg	66.41 (8.45)	65.81 (8.31)	69.78 (8.39)	<0.001
Hemoglobin, g/L	139.76 (17.51)	139.24 (17.68)	142.68 (16.24)	<0.001
White blood cell, 10^9/L	6.40 (1.44)	6.39 (1.45)	6.44 (1.42)	<0.001
ALT, U/L	21.07 (9.02)	20.91 (8.99)	21.97 (9.10)	<0.001
AST, U/L	22.27 (6.51)	22.18 (6.50)	22.81 (6.57)	<0.001

SCr, μmol/L	66.08 (17.64)	65.94 (17.66)	66.92 (17.49)	<0.001
TC, mmol/L	4.07 (0.87)	4.02 (0.85)	4.34 (0.90)	<0.001
HDL-C, mmol/L	1.31 (0.33)	1.31 (0.33)	1.31 (0.34)	<0.001
LDL-C, mmol/L	2.32 (0.75)	2.29 (0.74)	2.48 (0.78)	<0.001
Hepatic steatosis (%)				<0.001
no	999,729 (96.3)	854,291 (97.03)	145,438 (92.19)	
yes	38,441 (3.7)	26,113 (2.97)	12,328 (7.81)	
Type 2 diabetes (%)				<0.001
no	1,002,725(96.59)	858,333 (97.49)	144,392 (91.52)	
yes	35,445 (3.41)	22,071 (2.51)	13,374 (8.48)	
FH of hypertension (%)				<0.001
no	952605 (91.76)	802999 (91.21)	149606 (94.83)	
yes	85565 (8.24)	77405 (8.79)	8160 (5.17)	

Note: Data are presented as mean (standard deviation) or n (%).

Abbreviations: WC, waist circumference; BMI, body mass index; SBP, Systolic Blood Pressure; DBP, diastolic blood pressure; ALT, alanine aminotransferase; AST, aspartate transaminase; SCr, serum creatinine; TC, total cholesterol; HDL-C, high-density lipoprotein cholesterol; LDL-C, low-density lipoprotein cholesterol; FH of hypertension, family history of hypertension.
