# Supplementary material for: Two-Year Hypertension Incidence Risk Prediction in Populations in the Desert Regions of Northwest China: Prospective Cohort Study
Source: J Med Internet Res. 2025 Mar 12;27:e68442. doi: 10.2196/68442 (PMC11947627; doi:10.2196/68442)
Supplement: Multimedia Appendix 5 [file jmir_v27i1e68442_app5.pdf]

Multimedia Appendix 5. Baseline characteristics of the study population in prospective cohort

Characteristics	Overall	Non-HTN	HTN	p-value
	961,519	860,442	101,077	
Age at baseline, years	42.77 (14.45)	41.35 (13.96)	54.87 (12.71)	<0.001
Sex (%)				<0.001
male	431,824 (44.91)	380,602 (44.23)	51,222 (50.68)	
female	529,695 (55.09)	479,840 (55.77)	49,855 (49.32)	
Residence (%)				<0.001
rural	865,464 (90.01)	780,051 (90.66)	85,413 (84.5)	
urban	96,055 (9.99)	80,391 (9.34)	15,664 (15.5)	
WC, cm	85.00 (11.21)	84.54 (11.05)	88.98 (11.82)	<0.001
BMI, kg/m^2	24.64 (3.82)	24.47 (3.77)	26.07 (3.97)	<0.001
Educational level (%)				<0.001
illiterate or semi-literate	40,876 (4.25)	33,020 (3.84)	7,856 (7.77)	
primary school	376,961 (39.2)	323,076 (37.55)	53,885 (53.31)	
junior middle school	410,493 (42.69)	381,561 (44.34)	28,932 (28.62)	
senior middle school	85,923 (8.94)	79,533 (9.24)	6,390 (6.32)	
college degree and above	47,266 (4.92)	43,252 (5.03)	4,014 (3.97)	
Exercise frequency (%)				<0.001
never	922,358 (95.93)	826,725 (96.08)	95,633 (94.61)	
occasionally	16,839 (1.75)	14,565 (1.69)	2,274 (2.25)	
often	22,322 (2.32)	19,152 (2.23)	3,170 (3.14)	
Dietary patterns (%)				<0.001
meat and vegetable balance	936,106 (97.36)	838,706 (97.47)	97,400 (96.36)	
meat based	13,578 (1.41)	11,634 (1.35)	1,944 (1.92)	
vegetarian based	11,835 (1.23)	10,102 (1.17)	1,733 (1.71)	
Smoking status (%)				<0.001
never	861,763 (89.63)	770,604 (89.56)	91,159 (90.19)	
smoking	96,374 (10.02)	87,004 (10.11)	9,370 (9.27)	
quit smoking	3,382 (0.35)	2,834 (0.33)	548 (0.54)	
Alcohol intake (%)				<0.001
never	894,674 (93.05)	800,129 (92.99)	94,545 (93.54)	
occasionally	60,485 (6.29)	54,582 (6.34)	5,903 (5.84)	
often	6,360 (0.66)	5,731 (0.67)	629 (0.62)	
Heart rate, bpm	74.30 (9.41)	74.21 (9.40)	75.09 (9.49)	<0.001
SBP, mmHg	111.55 (11.66)	110.78 (11.52)	118.13 (10.69)	<0.001
DBP, mmHg	67.81 (8.01)	67.47 (7.96)	70.66 (7.82)	<0.001
Hemoglobin, g/L	141.38 (17.47)	141.05 (17.52)	144.17 (16.76)	<0.001
White blood cell, 10^9/L	6.44 (1.49)	6.43 (1.49)	6.55 (1.50)	<0.001
ALT, U/L	20.94 (9.20)	20.83 (9.20)	21.88 (9.16)	<0.001
AST, U/L	21.90 (6.81)	21.83 (6.81)	22.43 (6.74)	<0.001
SCr, μmol/L	66.59 (17.78)	66.52 (17.80)	67.24 (17.54)	<0.001

TC, mmol/L	4.14 (0.90)	4.11 (0.89)	4.41 (0.92)	<0.001
HDL-C, mmol/L	1.28 (0.34)	1.28 (0.34)	1.28 (0.35)	<0.001
LDL-C, mmol/L	2.25 (0.84)	2.22 (0.84)	2.42 (0.88)	<0.001
Hepatic steatosis (%)				<0.001
no	932,220 (96.95)	839,327 (97.55)	92,893 (91.9)	
yes	29,299 (3.05)	21,115 (2.45)	8,184 (8.1)	
Type 2 diabetes (%)				<0.001
no	921,947 (95.88)	830,612 (96.53)	91,335 (90.36)	
yes	39,572 (4.12)	29,830 (3.47)	9,742 (9.64)	
FH of hypertension (%)				<0.001
no	883,277 (91.86)	788,391 (91.63)	94,886 (93.87)	
yes	78,242 (8.14)	72,051 (8.37)	6,191 (6.13)	

Note: Data are presented as mean (standard deviation) or n (%).

Abbreviations: WC, waist circumference; BMI, body mass index; SBP, Systolic Blood Pressure; DBP, diastolic blood pressure; ALT, alanine aminotransferase; AST, aspartate transaminase; SCr, serum creatinine; TC, total cholesterol; HDL-C, high-density lipoprotein cholesterol; LDL-C, low-density lipoprotein cholesterol; FH of hypertension, family history of hypertension.
