# Supplementary material for: Two-Year Hypertension Incidence Risk Prediction in Populations in the Desert Regions of Northwest China: Prospective Cohort Study
Source: J Med Internet Res. 2025 Mar 12;27:e68442. doi: 10.2196/68442 (PMC11947627; doi:10.2196/68442)
Supplement: Multimedia Appendix 6 [file jmir_v27i1e68442_app6.pdf]

Multimedia Appendix 6. Evaluation of multiple machine learning models to predict the two-year hypertension incidence risk

Model	AUROC	AP	Acc	Precision	Recall
Logistic Regression	0.858(0.856-0.859)	0.518(0.514-0.523)	0.794(0.792-0.795)	0.408(0.405-0.411)	0.793(0.789-0.796)
Random Forest	0.864(0.862-0.866)	0.556(0.551-0.561)	0.870(0.869-0.871)	0.657(0.651-0.663)	0.303(0.298-0.307)
LightGBM	0.868(0.866-0.869)	0.569(0.565-0.574)	0.795(0.793-0.796)	0.410(0.407-0.414)	0.807(0.803-0.810)
XGBoost	0.868(0.866-0.870)	0.573(0.569-0.578)	0.796(0.795-0.797)	0.412(0.409-0.415)	0.804(0.801-0.808)
CatBoost	0.887(0.886-0.889)	0.609(0.604-0.613)	0.804(0.803-0.805)	0.425(0.422-0.428)	0.826(0.823-0.830)
FT-Transformer	0.864(0.862-0.866)	0.559(0.555-0.564)	0.869(0.868-0.871)	0.603(0.598-0.608)	0.411(0.407-0.416)
SAINT	0.867(0.865-0.869)	0.570(0.565-0.574)	0.871(0.870-0.872)	0.618(0.613-0.624)	0.391(0.386-0.395)
