# Supplementary material for: Two-Year Hypertension Incidence Risk Prediction in Populations in the Desert Regions of Northwest China: Prospective Cohort Study
Source: J Med Internet Res. 2025 Mar 12;27:e68442. doi: 10.2196/68442 (PMC11947627; doi:10.2196/68442)
Supplement: Multimedia Appendix 8 [file jmir_v27i1e68442_app8.pdf]

Multimedia Appendix 7. Statistical Significance Analysis of AUROC Comparisons Between CatBoost and Other Models

Model	Z-statitic	P-value[1]	P-value[2]
Logistic Regression	27.718	4.23E-169	2.66E-168
Random Forest	18.816	5.62E-79	1.18E-78
LightGBM	18.479	3.06E-76	4.83E-76
XGBoost	15.68	2.08E-55	2.19E-55
FT-Transformer	18.816	5.62E-79	1.18E-78
SAINT	16.464	6.69E-61	8.43E-61
