# Supplementary material for: Two-Year Hypertension Incidence Risk Prediction in Populations in the Desert Regions of Northwest China: Prospective Cohort Study
Source: J Med Internet Res. 2025 Mar 12;27:e68442. doi: 10.2196/68442 (PMC11947627; doi:10.2196/68442)
Supplement: Multimedia Appendix 9 [file jmir_v27i1e68442_app9.pdf]

Multimedia Appendix 9. Classification of individuals into four risk levels based on PPV in the prospective dataset

Risk category intervals	Low [0, 0.15]	Medium [0.15, 0.3]	High [0.3, 0.8]	very High [0.8,1]	Total
Total, n	570,742	131,598	245,204	13,975	961,519
Case, n	16,392	16,661	62,283	5,741	101,077
PPV	0.000	0.127	0.254	0.411	
Acc	0.971	0.134	0.254	0.411	
