# Supplementary material for: Two-Year Hypertension Incidence Risk Prediction in Populations in the Desert Regions of Northwest China: Prospective Cohort Study
Source: J Med Internet Res. 2025 Mar 12;27:e68442. doi: 10.2196/68442 (PMC11947627; doi:10.2196/68442)
Supplement: Multimedia Appendix 10 [file jmir_v27i1e68442_app10.pdf]

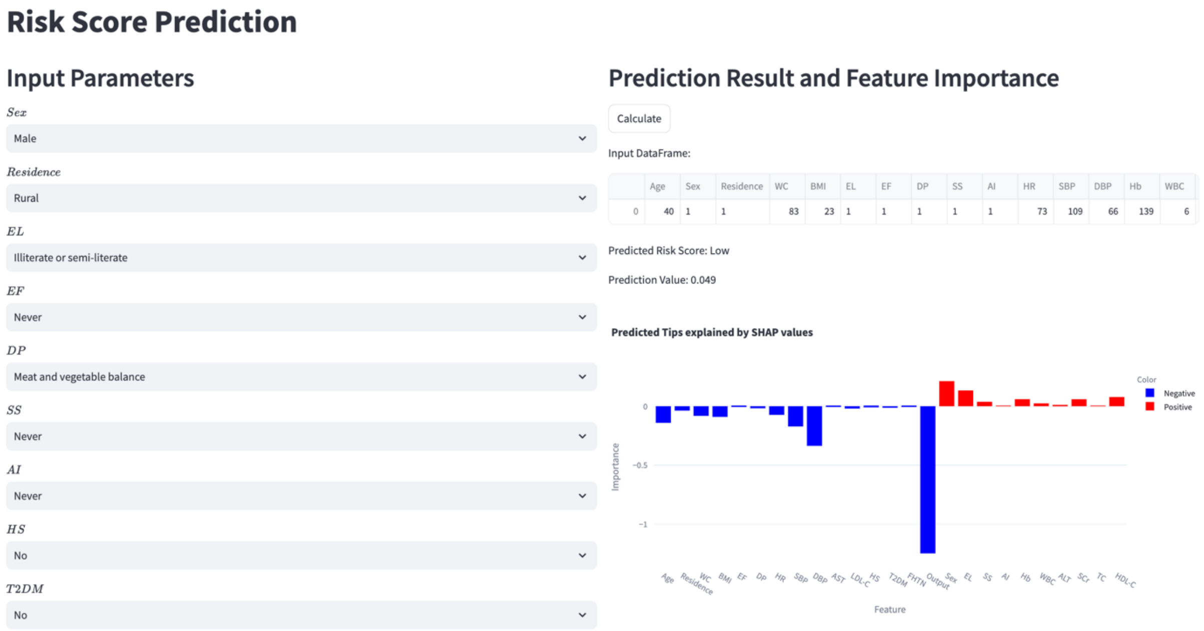

Multimedia Appendix 10. Main interface of the web-based application for two-year hypertension incidence risk prediction
